# Leveraging artificial intelligence to predict ERG gene fusion status in prostate cancer

**DOI:** 10.1186/s12885-022-09559-4

**Published:** 2022-05-05

**Authors:** Vipulkumar Dadhania, Daniel Gonzalez, Mustafa Yousif, Jerome Cheng, Todd M. Morgan, Daniel E. Spratt, Zachery R. Reichert, Rahul Mannan, Xiaoming Wang, Anya Chinnaiyan, Xuhong Cao, Saravana M. Dhanasekaran, Arul M. Chinnaiyan, Liron Pantanowitz, Rohit Mehra

**Affiliations:** 1 Department of Pathology, University of Michigan Medical School, Ann Arbor, MI USA; 2 Department of Pathology and Laboratory Medicine, Jackson Memorial Hospital, Miami, FL USA; 3 Department of Pathology, Vanderbilt University Medical Center, Nashville, TN USA; 4 Department of Urology, University of Michigan Medical School, Ann Arbor, MI USA; 5 Department of Radiation Oncology, University Hospitals Seidman Cancer Center, Case Western Reserve University School of Medicine, Cleveland, OH USA; 6 Department of Medical Oncology, University of Michigan Medical School, Ann Arbor, MI USA; 7 Michigan Center for Translational Pathology, Ann Arbor, MI USA; 8 Rogel Cancer Center, Michigan Medicine, Ann Arbor, MI USA; 9 Howard Hughes Medical Institute, Ann Arbor, MI USA

**Keywords:** Prostate cancer, ERG, Deep learning, Artificial intelligence, Gene fusion, Adenocarcinoma, Whole slide imaging

## Abstract

**Background:**

*TMPRSS2-ERG* gene rearrangement, the most common E26 transformation specific (ETS) gene fusion within prostate cancer, is known to contribute to the pathogenesis of this disease and carries diagnostic annotations for prostate cancer patients clinically. The *ERG* rearrangement status in prostatic adenocarcinoma currently cannot be reliably identified from histologic features on H&E-stained slides alone and hence requires ancillary studies such as immunohistochemistry (IHC), fluorescent in situ hybridization (FISH) or next generation sequencing (NGS) for identification.

**Methods:**

**Objective:**

We accordingly sought to develop a deep learning-based algorithm to identify *ERG* rearrangement status in prostatic adenocarcinoma based on digitized slides of H&E morphology alone.

**Design:**

*Setting, and Participants:* Whole slide images from 392 in-house and TCGA cases were employed and annotated using QuPath. Image patches of 224 × 224 pixel were exported at 10 ×, 20 ×, and 40 × for input into a deep learning model based on MobileNetV2 convolutional neural network architecture pre-trained on ImageNet. A separate model was trained for each magnification. Training and test datasets consisted of 261 cases and 131 cases, respectively. The output of the model included a prediction of ERG-positive (ERG rearranged) or ERG-negative (ERG not rearranged) status for each input patch.

*Outcome measurements and statistical analysis:* Various accuracy measurements including area under the curve (AUC) of the receiver operating characteristic (ROC) curves were used to evaluate the deep learning model.

**Results and Limitations:**

All models showed similar ROC curves with AUC results ranging between 0.82 and 0.85. The sensitivity and specificity of these models were 75.0% and 83.1% (20 × model), respectively.

**Conclusions:**

A deep learning-based model can successfully predict *ERG* rearrangement status in the majority of prostatic adenocarcinomas utilizing only H&E-stained digital slides. Such an artificial intelligence-based model can eliminate the need for using extra tumor tissue to perform ancillary studies in order to assess for *ERG* gene rearrangement in prostatic adenocarcinoma.

## Introduction

In medicine today being driven by cutting-edge cancer therapeutics and a personalized approach to delivering healthcare, artificial intelligence (AI) has had an additive impact on the digital transformation in the field. An increasing role of AI, including machine and deep learning methods, is being applied not only to diverse ‘omics’ fields such as genomics, pharmacogenomics, and proteomics, but also conventional clinical medicine disciplines such as radiology, pathology, immuno-oncology, and others. Substantial published data is accruing indicating that AI can improve diagnoses, offer predictions (e.g. correlation with underlying tumor genomics, theranostic response to treatment), and render prognostic information employing only image data. In the field of genitourinary medicine, deep learning models have been developed to reliably subtype renal cell carcinomas [1] and aid pathologists in the automation of prostate biopsy interpretation [2].

Prostate cancer continues to be a major global cause of morbidity and mortality, with a significant death rate within the United States. The current National Comprehensive Cancer Network (NCCN) guidelines have successfully incorporated genomic tools to guide and improve prostate cancer management. In terms of prostate cancer biology, the discovery of recurrent gene fusions in a majority of prostate cancers has had important clinical and biological implications with a paradigm shift in our understanding of the genomics of common epithelial tumors. Several years ago, our group identified genomic rearrangements in prostate cancer resulting in the fusion of the 5’ untranslated end of *TMPRSS2* (Transmembrane serine protease 2; a prostate-specific gene controlled by androgen) to members of the ETS (E26 transformation-specific) family of oncogenic transcription factors, leading to the over-expression of ETS genes like *ERG* (ETS-related gene), *ETV1* (ETS variant transcription factor 1), *ETV4* (ETS variant transcription factor 4), and others, with *ERG* being the most common gene fusion partner [3]. ETS gene fusions are found within a distinct class of prostate cancers that are associated with diagnosis, prognosis, and targeted therapy [4, 5].

Almost half of all prostatic adenocarcinomas, including clinically localized as well as metastatic tumors, are associated with *TMPRSS2*-*ERG* gene fusion [6]. This causes juxtaposition of the *ERG* gene to androgen-responsive regulatory elements of *TMPRSS2*, that leads to aberrant androgen receptor driven over-expression of ERG protein. Currently, the gold standard for detection of *ERG* gene rearrangement is fluorescent in situ hybridization (FISH) or next generation sequencing (NGS) technology. In routine clinical and surgical pathology practice, while some histologic features associated with prostatic adenocarcinoma such as blue mucin production and prominent nucleoli in tumor cells have been shown to demonstrate association with underlying *ERG* gene rearrangement [7]. However, these morphologic features are not consistently present in the tumors with *ERG* gene rearrangement and by themselves they are not reliably predictive, hence detection of over-expression of *ERG* protein by immunohistochemistry (IHC) is often used as a surrogate to identify *ERG* gene rearrangement in prostate cancer.

Recent studies have proven largely successful at leveraging AI-based models to recognize and characterize prostate cancer on whole slide imaging (WSI) [8–13]. The training strategies for the AI-models fall under one of two main categories: supervised learning, or weakly supervised learning. The first strategy requires that annotations be made at the pixel-level for each whole slide image. While this approach is meticulous and often arduous for the expert who is annotating the case, it benefits from lower computational burden and fewer overall case requirements for training compared to the latter strategy. However, whenever possible, the weakly supervised deep learning strategy is often preferred due to lower burden on the expert pathologist annotator since this method only requires slide-level annotations i.e. one annotation per slide [14]. The most notable example of a successful weakly supervised deep learning AI-model was developed by Campanella et al. and has gone on to receive the first ever FDA approval for an AI product in Digital Pathology [13].

Morphologic characterization is not the only area in which AI-based models have been successful. Much advancement has been made in predicting genetic mutations based on AI-driven histomorphologic analysis. Deep learning has been used to classify and predict common mutations in a variety of tumors including non-small cell lung carcinomas, bladder urothelial carcinomas, renal cell carcinoma and melanomas. All these methods are based on AI analysis of histology images alone without additional information [15–18]. Given the success of these models, it is feasible that similar deep learning algorithms could be developed to potentially predict underlying genetic aberrations in prostate adenocarcinoma from histopathologic images.

In this study, we accordingly utilized Hematoxylin and Eosin (H&E)-stained whole slide images (WSIs) of prostate adenocarcinoma and sought to develop a deep learning algorithm that could distinguish *ERG* rearranged prostate cancers from those without *ERG* rearrangement. Our results suggest that image features alone can analyze subtle morphological differences between *ERG* gene fusion positive and negative prostate cancers, which would thereby eliminate the need to utilize extra tumor tissue to perform ancillary studies such as IHC, FISH or NGS testing in order to assess for *ERG* gene rearrangement.

## Materials and methods

### Data acquisition and slide scanning

Patient samples were procured from Michigan Medicine and the study was performed under Institutional Review Board-approved protocols. A retrospective pathological and clinical review of radical prostatectomies performed between November 2019 and August 2021 at the University of Michigan Health System was conducted. Only patients without prior history of treatment were included. A total of 163 patients were randomly selected for analysis. Electronic medical records and pathology reports were reviewed to analyze clinical parameters (age at diagnosis, PSA level at diagnosis and treatment modality), and pathological variables (Gleason Score/Grade Group). The H&E-stained glass slides from all cases were re-reviewed by two genitourinary pathologists (VD and RoM) to confirm the diagnosis and evaluate morphologic features. Gleason Score, where applicable, was assigned according to the 2014 modified Gleason grading system and Grade Group was assigned according to the established criteria endorsed by the World Health Organization (WHO). A representative H&E glass slide from each radical prostatectomy was scanned using an Aperio AT2 scanner using 20 × objective (Leica Biosystems Inc., Buffalo Grove, IL, USA and 40 × magnification (0.25 μm/pixel resolution) was achieved using a 2 × optical magnification changer. The scanner generated WSIs as svs file format. The microscopic photographs were obtained using an Olympus BX43 microscope with attached camera (Olympus DP47) and cellSens software. *ERG* rearrangement status for in-house cases was determined by IHC as described below. Out of 163 evaluated cases, 6 cases showed heterogeneity of ERG staining and were hence excluded from further analysis. The remaining 157 in-house cases were included in the final dataset utilized for the purposes of this study.

From the cancer genome atlas (TCGA) database (https://portal.gdc.cancer.gov), we downloaded a total of 300 formalin fixed paraffin embedded H&E WSIs of prostate cancer. The images without any discernible cancer morphologically were excluded, and for final analysis 242 images from 235 patients were included. These TCGA prostate cancer cases were included because they have a known *ERG* rearrangement status, confirmed from previously reported genomic studies [19].

### Immunohistochemistry

IHC was performed on sections that are selected for scanning for all in-house cases using anti-ERG rabbit monoclonal antibodies (EPR3864, Ventana, prediluted). Appropriate positive and negative controls were included. ERG immunohistochemical expression was performed based on clinically used evaluation criterion where expression of ERG protein within a tumor focus was considered to be positive and such a tumor focus was designated as ERG-positive. Tumor foci which do not expression ERG protein were designated as ERG-negative.

### Deep learning model architecture and evaluation

**Fig. 1:**
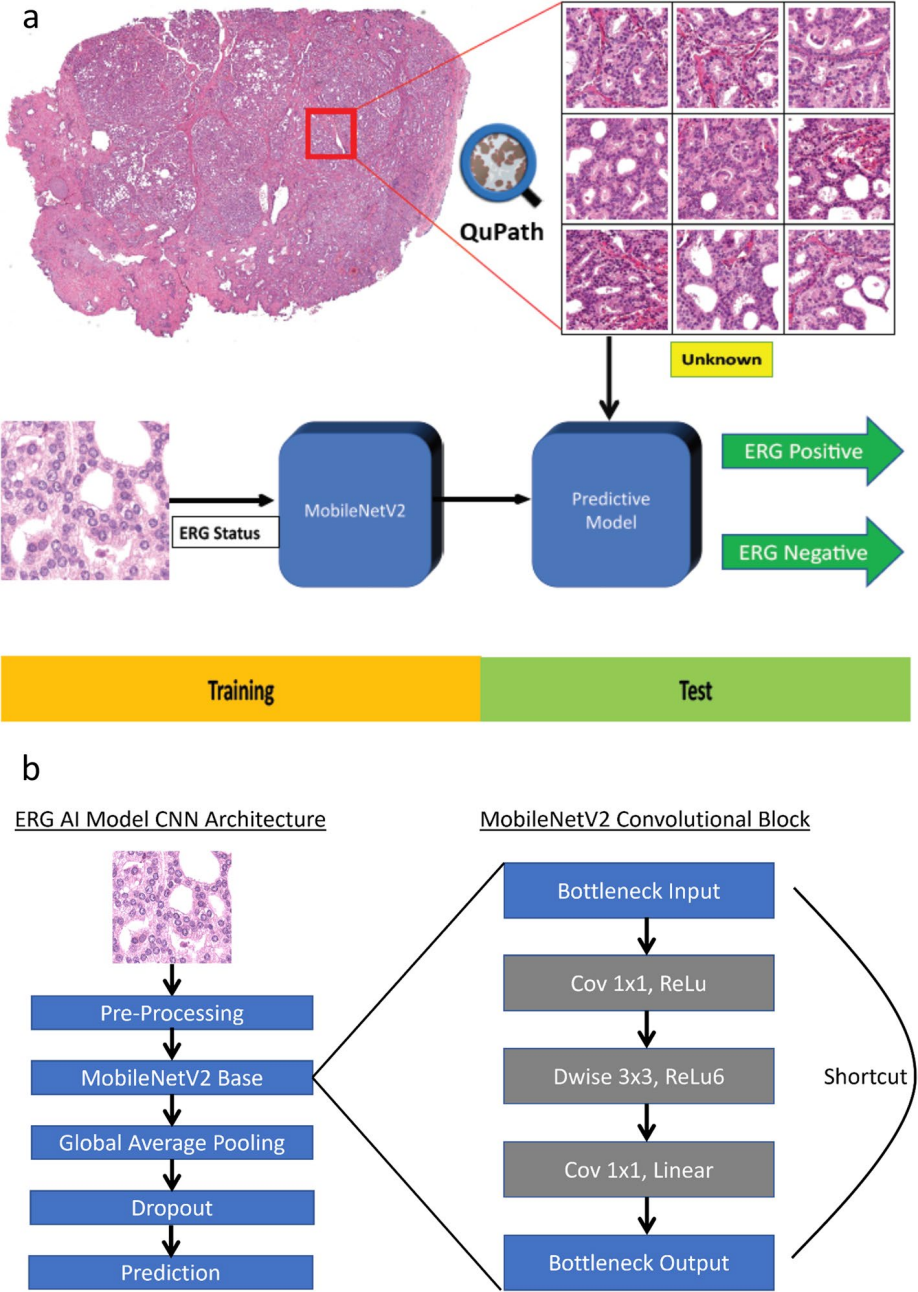
Workflow schematic summarizing our algorithm development. **a** (Top panel) Whole slide images of H&E-stained prostate adenocarcinoma resections were spilt using QuPath into many 224 × 224 pixel patches for input into a convolutional neural network (CNN). Unknown yellow box indicates a separate subset of cases not included as part of the training subset. (Bottom panel) Patches labeled with ERG status were used for CNN training utilizing MobileNetV2. Final prediction of patches into ERG-negative or ERG-positive was based on highest probability. **b** MobileNetV2 convolutional block structure (adapted from Sandler et al.)

Regions of tumor from WSIs were manually annotated using QuPath v0.2.3 [20]. The regions annotated as either ERG-positive or ERG-negative were exported as 224 × 224 pixel sized JPEG image patches at 10 ×, 20 ×, and 40 × magnifications, for input into the deep learning model. For all magnifications image patches were taken from same area. All 235 TCGA cases and 26 in-house cases (*n* = 261, 67%) were used for training purposes. A separate hold out test dataset, that included the remaining in-house cases (*n* = 131, 33%), were used for performance evaluation of the model (Table 1). A total of 763,945 patches were generated from regions of interest for the training set and 264,688 patches were generated for the hold-out test set. Patches from the training sets were further randomly subdivided into training, validation, and test subsets with a split ratio of 80:16:4, respectively. Using the Python Keras Application Programming Interface (API), we developed a deep learning algorithm for distinguishing between ERG rearranged and ERG non-rearranged prostate cancer. Development and testing was performed using a computer equipped with an NVIDIA RTX 2070 Super graphics processing unit (GPU) and 16 GB of RAM at 3200 MHz. The algorithm is based on the MobileNetV2 convolutional neural network (CNN) architecture pre-trained on ImageNet. The pre-trained MobileNetV2 network was used as the base model. It is preceded by a pre-processing layer which scales input pixel values between -1 and 1 for MobileNetV2. Subsequent to the base model, an additional global average layer, dropout layer, and prediction layer were added. Given the binary nature of this classification task, the prediction layer consisted of a dense layer with a sigmoid activation function. Three different models were trained for each of the different image magnifications (10 ×, 20 ×, and 40 ×). Model weights were fine-tuned using ERG-positive and ERG-negative labeled H&E patches. Data augmentation techniques consisting of horizontal flips, vertical flips, rotations, and contrast variations were applied to the input patches during the training process. After initial training, the hyperparameters were further adjusted using validation set. Finally, models were evaluated using hold-out test set cases, which were independent of training and validation sets, with unlabeled patches as inputs. The output of the models consisted of a prediction of ERG-positive or ERG-negative for each input patches. The workflow used in developing our algorithm is summarized in Fig. 1.

**Table 1:** Distribution of training and hold-out test datasets utilized for algorithm development

Dataset	Subset	Patients	ERG status		Gleason grade group				
			Positive	Negative	1	2	3	4	5
TCGA cohort	Training set	235	123(52%)	112(48%)	41	68	60	32	34
Internal cohort	Training set	26	11(42%)	15(58%)	1	17	6	0	2
	Hold-out test set	131	60(46%)	71(54%)	0	67	31	4	29

Training subset includes initial training, cross-validation and testing sets. Hold-out test set refers to a separate subset of cases not included as part of the training subset. *TCGA* The Cancer Genome Atlas

## Results

**Fig. 2:**
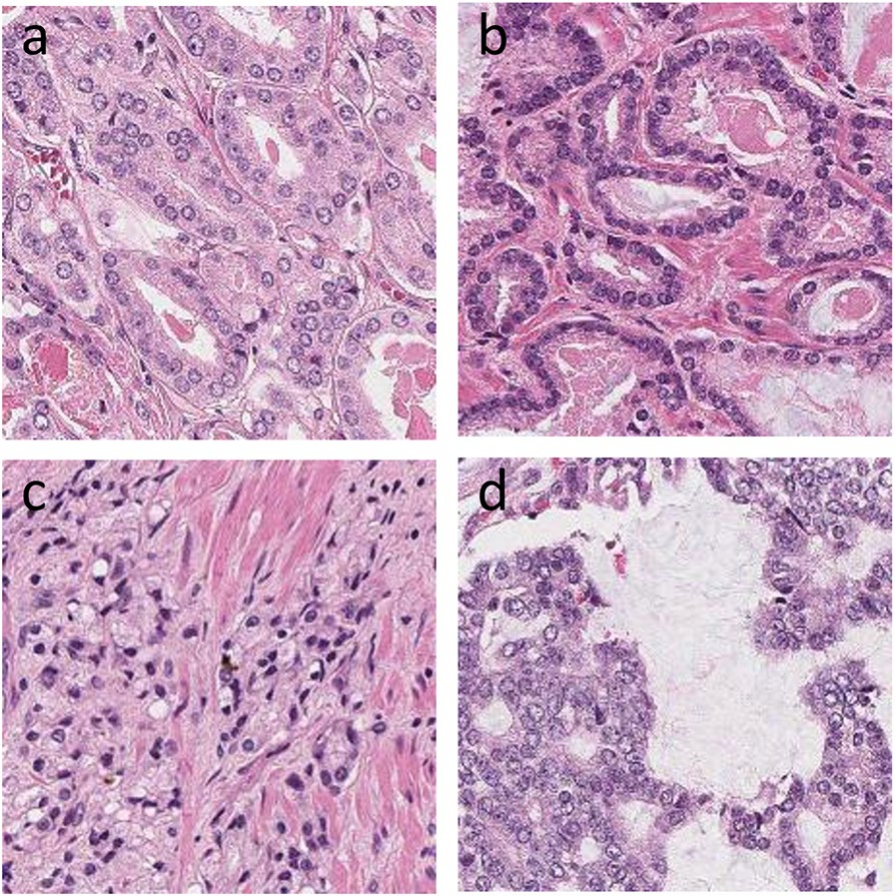
Patches as classified by AI algorithm. **a** ERG-negative low grade (100 ×). **b** ERG-positive low grade (100 ×). **c** ERG-negative high grade (100 ×). **d** ERG-positive high grade (100 ×)

In this study we developed an AI-based algorithm to establish whether *ERG* gene arrangement can be determined solely from H&E-based histologic images of patients with prostate adenocarcinoma. A total of 26 in-house prostatectomy cases and 235 cases obtained from the TCGA prostate cancer cohort were used for initial training of the model and a separate hold-out test cohort of 131 in-house cases was used for model evaluation (Table 1). The analysis was performed employing 10 ×, 20 × and 40 × image magnifications. An overall diagnosis for each WSI was assigned based on percentage of labelled patches favoring an ERG-positive or ERG-negative diagnosis. For each magnification, a cut-off of proportion of ERG positive patches were determined that gives best accuracy. A WSI was labeled as ERG-positive if a proportion of the ERG positive patches were greater than the cut-off determined at a particular magnification. For performance metrics, each WSI was defined as follows: true positive is defined as the correct prediction of ERG-positive cases, false negative as incorrect prediction of ERG-positive cases, true negative as correct prediction of ERG-negative cases, and false positive as incorrect prediction of ERG-negative cases. Representative patches as identified by the algorithm are provided in Fig. 2.

**Fig. 3:**
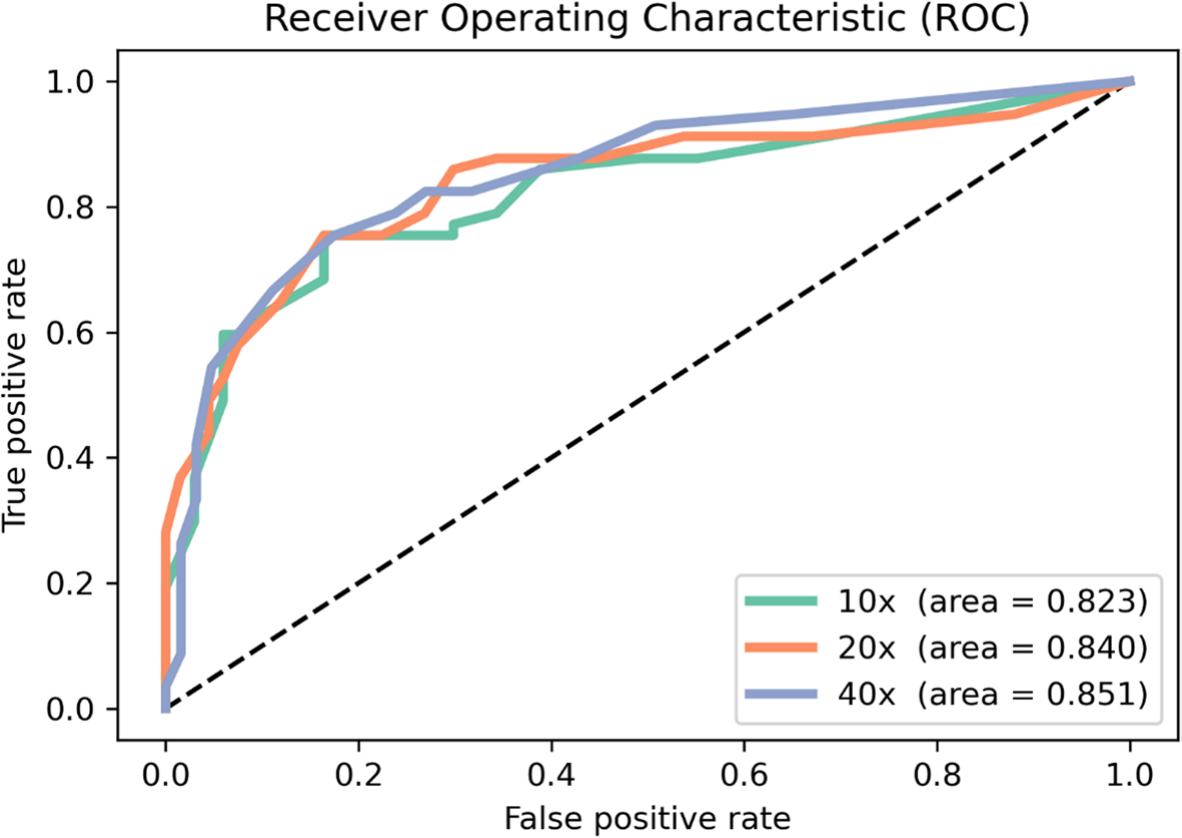
Receiver operator characteristics (ROC) and area under curve for models at different magnifications (10x, 20 × and 40x)

ROC curves were generated for all three models. The best accuracy of 79.4% and an area under the ROC curve of 0.85 were achieved at 20 × and 40 × magnification, respectively (Fig. 3 and Table 2). A total of 104 out of 131 cases (79.4%) were identified correctly by the AI-based algorithm, with a sensitivity of 75.0%, specificity of 83.1%, positive predictive value (PPV) of 78.9%, and negative predictive value (NPV) of 79.7%. The performance of the algorithm at all magnifications was almost equivalent, yielding an accuracy in the range of 78.6% and 79.4% as well as area under the ROC curve in the range of 0.82 and 0.85. The cut-off for ERG-positive patches was 0.5, where best accuracy was achieved for 20 × magnification.

**Table 2:** Performance metrics of AI-based models at different magnifications

Magnification	AUC	TP	FP	TN	FN	Sensitivity	Specificity	PPV	NPV	Accuracy	F1 score	Cut-off
10 ×	0.82	45	13	58	15	75.0%	81.7%	77.6%	79.5%	78.6%	0.78	0.4
20 ×	0.84	45	12	59	15	75.0%	83.1%	78.9%	79.7%	79.4%	0.79	0.5
40 ×	0.85	45	13	58	15	75.0%	81.7%	77.6%	79.5%	78.6%	0.78	0.35

*AUC* Area under curve in receiver operator characteristics curve, *TP* True positive (correctly classified as ERG-positive), *FP* False positive (incorrectly classified as ERG-positive), *TN* True negative (correctly classified as ERG-negative), *FN* False negative (incorrectly classified as ERG-negative), *PPV* Positive predictive value, *NPV* Negative predictive value, Cut-off indicates cut-off value of proportion of positive patches that gives best accuracy

The morphologic features of prostatic adenocarcinoma vary greatly according to the grade of the tumor. Hence, we sought to evaluate performance of our model according to different Gleason grades. In order to assess the performance of our model according to different Gleason grades, we evaluated our algorithm separately for subgroups based on their assigned Grade Groups within this cohort. Hold-out cases were categorized into low-grade and high-grade tumors; the lower-grade tumors included Grade Groups 1 and 2, of which these tumors were predominantly comprised of a Gleason grade 3 component; in contrast, the higher-grade tumors were comprised of Grade Groups 3 and higher with the majority of these tumors comprised of Gleason grade 4 and 5 components. With Gleason grades taken into consideration, equivalent accuracy were obtained at all magnifications. For lower-grade tumors the accuracy achieved was 86.6% at 10 × and 20 × magnifications, while accuracy for higher-grade tumors was 73.4% at 40 × magnification. These results are summarized in Table 3.

**Table 3:** Algorithm performance metrics based on tumor grade

Magnification	Grade Group	TP	FP	TN	FN	Sensitivity	Specificity	PPV	NPV	Accuracy	F1 score	Cut-off
10 ×	1–2	33	8	25	1	97.1%	75.8%	80.5%	96.2%	86.6%	0.88	0.4
	3–5	12	5	33	14	46.2%	86.8%	70.6%	70.2%	70.3%	0.56	0.4
20 ×	1–2	28	3	30	6	82.4%	90.9%	90.3%	83.3%	86.6%	0.86	0.5
	3–5	17	9	29	9	65.4%	76.3%	65.4%	76.3%	71.9%	0.65	0.5
40 ×	1–2	28	5	28	6	82.4%	84.8%	84.8%	82.4%	83.6%	0.84	0.35
	3–5	17	8	30	9	65.4%	78.9%	68.0%	76.9%	73.4%	0.67	0.35

*TP* True positive (correctly classified as ERG-positive), *FP* False positive (incorrectly classified as ERG-positive), *TN* True negative (correctly classified as ERG-negative), *FN* False negative (incorrectly classified as ERG-negative), *PPV* Positive predictive value, *NPV*: Negative predictive value, Cut-off indicates cut-off value of proportion of positive patches that gives best accuracy

## Discussion

IHC has become the workhorse of molecular phenotyping for tissues and currently serves as a reliable surrogate to actually performing expensive molecular testing. However, IHC is time-consuming, can be expensive, and dependent on appropriate tissue handling procedures, reagents, and expert laboratory technicians. Furthermore, immunostain findings require visual inspection using a microscope and thus depend on the subjective interpretation of pathologists [21, 22]. Recent technological progress in digital pathology and AI has shown that these new modalities can be used to not only improve efficiency of pathologists, but also provide comparable diagnostic accuracy to pathologists employing traditional light microscopy [12]. Within the domain of surgical pathology, AI-based algorithms can analyze digitized histomorphologic features to effectively distinguish neoplastic and non-neoplastic lesions [23, 24], detect metastasis in lymph nodes [25], predict genomic fusion status within renal neoplasms [26], subtype renal tumors [1], detect prostate cancer in biopsy material [27], as well as grade aggressiveness of certain tumors [4]. To date, AI-based studies have been applied to prostate cancer pathology to assist with diagnosis, Gleason grading, prognosis, as as well as predict underlying molecular aberrations such as phosphatase and tensin homology (PTEN) loss [2, 28, 29]. In the present study, we developed a deep learning model to predict *ERG* rearrangement status in patients with prostatic adenocarcinoma. To the best of our knowledge, this is the first study to identify this genomic status directly from scanned H&E-stained slides in surgically resected prostate cancer cases.

*TMPRSS2*-*ERG* rearrangement, the most common ETS gene fusion in prostate cancer, brings *ERG* expression under androgen control via androgen receptor-mediated *TMPRSS2* regulation and results in over-expression of ERG protein [3]. Microscopically, while some *ERG* rearranged prostate cancers are enriched with features such as intraluminal blue mucin and prominent nucleoli, the spectrum of morphology is quite variable and inconsistently predictive of the presence of an *ERG* rearrangement at the genomic level [7]. Hence, it remains challenging to faithfully distinguish prostate cancer with *ERG* rearrangement from those with wild type *ERG* and other molecular subtypes based only on the microscopic evaluation of H&E-stained pathological tissues. As a result, *ERG* gene rearrangement status is usually confirmed by immunohistochemical identification of the overexpression of ERG protein or by dual-color break-apart FISH. However, these ancillary tests require additional time and resources, and they consume precious tissue.

In this study, we demonstrated that a digitized H&E-stained slide analyzed using a deep learning-based model can successfully predict *ERG* fusion status in the majority of prostate cancer cases. We believe that this algorithm can eliminate the need for using extra tumor tissue to perform lengthy and expensive ancillary studies to assess for *ERG* gene rearrangement in prostatic adenocarcinoma. Our AI model was able to accurately predict the presence of an *ERG* gene rearrangement in a large number of cases with varying morphologic patterns and Grade groups, including tumors with low-grade (Grade Group 2 or less) and high-grade features (Grade Group 3 or higher). Higher accuracy was seen in lower-grade tumors. One possibility for this observation may be that higher-grade tumors typically exhibit more diverse morphology.

*ERG* gene rearrangement is known to contribute to the pathogenesis of prostate cancer and provides important clues about the multifocality and metastatic dissemination of this disease. The specificity of this gene rearrangement in prostate cancer allows ERG evaluation by IHC to be of diagnostic value in both primary and metastatic tumors originating from the prostate [6, 30, 31]. *TMPRSS2*-*ERG* fusions are also prime candidates for the development of new diagnostic assays, including urine-based noninvasive assays [32]. There have been conflicting reports regarding the prognostic value of *ERG* gene rearrangement and its overexpression in prostatic cancer. Hägglöf et al. have demonstrated that high expression of *ERG* is associated with higher Gleason score, aggressive disease and poor survival rates [33]. Similarly, Nam et al. demonstrated that the *TMPRSS2-ERG* fusion gene predicts cancer recurrence after surgical treatment and that this prediction is independent of grade, stage and prostate specific antigen (PSA) levels in blood [34]. Mehra et al. demonstrated *ERG* rearrangement to be associated with a higher stage in prostate cancer [35]. A subsequent study by Fine et al. demonstrated a subset of prostate cancers with *TMPRSS2-ERG* copy number increase, with or without rearrangement, to be associated with higher Gleason score [36]. Nevertheless, other studies have found no association between *TMPRSS2-ERG* fusion and stage, grade, recurrence, or progression [37, 38]. Additionally, in the TCGA dataset, in our limited analyses, we did not see any other particular genetic mutation that is significantly different between *ERG* rearranged and non-*ERG* rearranged cases. Currently, there is no ERG-targeted therapy approved for treatment of prostate cancer. However, peptidomimetic targeting of transcription factor fusion products has been demonstrated to provide a promising therapeutic strategy for prostate cancer [39]. Previous *ERG* fusion driven biomarker clinical trials utilized interrogation of *ERG* rearrangement status employing IHC or FISH tests [40]. Our study provides a viable and inexpensive alternative to ancillary tissue-based testing methods to detect *ERG* rearrangement status in prostate cancer.

Our study has several strengths and potential limitations. Notable strengths include the use of H&E stained slides only (without the need for concurrent genomic investigation) to predict *ERG* gene fusion status in prostate cancer, utilization of WSI, and employment of diverse datasets including in-house and TCGA datasets with different H&E staining qualities to improve the robustness of our algorithm. This application carries strength in eliminating the need for complex molecular testing utilizing FISH, next-generation sequencing, or molecular surrogate assays like immunohistochemistry; utilizing of H&E slides only allows an easy, economical and efficient methodology to detect ERG gene rearrangement utilizing AI developed model. Importantly, our study paves a foundation for utilizing basic laboratory tools in assessing genomic rearrangements in diverse set of human malignancies (of prostate and other genitourinary tumors).

Computational limitations for both training and test set evaluation were considered when deciding which neural network architecture to utilize. Commonly used architectures for image classification tasks include Inception, VGG16, ResNet50, and MobileNet, among others. Each architecture comes with its own strengths and limitations and each one is designed to be optimal under specific circumstances. For example, the Inception architecture serves the purpose of reducing computational cost by implementing a shallower network compared to ResNet50 which may negatively impact computational accuracy. MobileNetV2, part of the MobileNet family, further addresses issues of size and speed and is optimally designed for mobile device applications which often require computationally limited platforms. This is accomplished by utilizing 19 inverted residual bottleneck layers following the initial fully convolutional layer, The 19 bottleneck layers are subsequently followed by a point convolutional layer, pooling average layer, and a final convolutional layer. Taking into consideration that these AI-applications are ultimately intended for clinical laboratory settings which may not have access to high-end computational hardware, we ultimately chose a MobileNetV2 architecture pre-trained on ImageNet as our base model due to its balance between accuracy and computational cost [41, 42].

Hardware limitations necessitating relatively small input tiles may contribute to our model’s performance. Our training set was relatively enriched in lower-grade tumors as high-grade cancers are less common in daily clinical urological practice. Follow-up studies incorporating more higher-grade tumors will be needed to better assess the performance of our AI-based tool in such scenario. Our algorithm was developed using resection specimens, and further studies would be needed to interrogate findings in biopsy specimens that display smaller volumes of tumor; as a consequence, cut-offs used in this study may need to be adjusted. For the purposes of this study, we did not address disease heterogeneity and multifocality; future studies are likely to address these phenomena.

## Conclusion

We demonstrated that *ERG* rearrangement status in prostate adenocarcinoma can be reliably predicted directly from H&E-stained digital slides utilizing a deep learning algorithm with high accuracy. This approach has great potential to automate digital workflows and avoid using tissue-based ancillary studies to assess for *ERG* gene rearrangement.

## Data Availability

The TCGA datasets are available in the public domain; rest is not applicable. The code for the model is deposited at https://doi.org/10.5281/zenodo.5911163.

## Abbreviations

AI: Artificial intelligence
AUC: Area under curve
ERG: ETS-related gene
ETS: E26 transformation-specific
ETV1: ETS variant transcription factor 1
ETV4: ETS variant transcription factor 4
FISH: Fluorescence in situ hybridization
H&E: Hematoxylin and eosin
IHC: Immunohistochemistry
IRB: Institutional Review Board
mCRPC: Metastatic castrate resistant prostate cancer
NCCN: National Comprehensive Cancer Network
NPV: Negative predictive value
PPV: Positive predictive value
PSA: Prostate specific antigen
PTEN: Phosphatase and tensin homolog
ROC: Receiver operating characteristic
TCGA: The Cancer Genome Atlas
TMPRSS2: Transmembrane serine protease 2
WHO: World Health Organization
WSI: Whole slide images
